# Exercise training improves quality of life in women with subclinical hypothyroidism: a randomized clinical trial

**DOI:** 10.20945/2359-3997000000073

**Published:** 2018-10-01

**Authors:** Francisco Zacaron Werneck, Emerson Filipino Coelho, Saulo Peters Almas, Marília Mendes do Nascimento Garcia, Heloina Lamha Machado Bonfante, Jorge Roberto Perrout de Lima, Patrícia dos Santos Vigário, Míriam Raquel Meira Mainenti, Patrícia de Fátima dos Santos Teixeira, Mário Vaisman

**Affiliations:** 1 Universidade Federal de Ouro Preto Universaidade Federal de Ouro Preto Ouro Preto MG Brasil Universaidade Federal de Ouro Preto (UFOP), Ouro Preto, MG, Brasil; 2 Universidade Federal do Rio de Janeiro Universidade Federal do Rio de Janeiro Rio de Janeiro RJ Brasil Universidade Federal do Rio de Janeiro (UFRJ), Rio de Janeiro, RJ, Brasil; 3 Universidade Federal de Juiz de Fora Universidade Federal de Juiz de Fora Juiz de Fora MG Brasil Universidade Federal de Juiz de Fora (UFJF), Juiz de Fora, MG, Brasil; 4 Faculdade de Ciências Médicas e da Saúde de Juiz de Fora Faculdade de Ciências Médicas e da Saúde de Juiz de Fora Juiz de Fora MG Brasil Faculdade de Ciências Médicas e da Saúde de Juiz de Fora (Suprema), Juiz de Fora, MG, Brasil; 5 Centro Universitário Augusto Motta Centro Universitário Augusto Motta Rio de Janeiro RJ Brasil Centro Universitário Augusto Motta (Unisuam), Rio de Janeiro, RJ, Brasil; 6 Escola de Educação Física do Exército Rio de Janeiro RJ Brasil Escola de Educação Física do Exército (EsEFEx), Rio de Janeiro, RJ, Brasil

**Keywords:** Hypothyroidism, quality of life, exercise training, functional capacity

## Abstract

**Objective:**

The aim was to evaluate the quality of life (HRQoL) in women with subclinical hypothyroidism (sHT) after 16 weeks of endurance training.

**Subjects and methods:**

In the first phase, a cross-sectional study was conducted in which 22 women with sHT (median age: 41.5 (interquartile range: 175) years, body mass index: 26.2 (8.7) kg/m^2^, thyroid stimulating hormone > 4.94 mIU/L and free thyroxine between 0.8 and 1.3 ng/dL were compared to a group of 33 euthyroid women concerned to HRQoL. In the second phase, a randomized clinical trial was conducted where only women with sHT were randomly divided into two groups: sHT-Tr (n = 10) - participants that performed an exercise program - and sHT-Sed (n = 10) - controls. Exercise training consisted of 60 minutes of aerobic activities (bike and treadmill), three times a week, for 16 weeks. The HRQoL was assessed by the SF-36 questionnaire in the early and at the end of four months.

**Results:**

Women with sHT had lower scores on functional capacity domain in relation to the euthyroid ones (770 ± 23.0 *vs.* 88.8 ± 14.6; p = 0.020). The sHT-Tr group improved functional capacity, general health, emotional aspects, mental and physical component of HRQoL after training period, while the sHT-Sed group showed no significant changes.

**Conclusion:**

After 16 weeks of aerobic exercise training, there were remarkable improvements in HRQoL in women with sHT.

## INTRODUCTION

Thyroid hormones (T_3_ and T_4_) act on most body cells, and changes in serum concentrations of these hormones cause impact on health of people (1). When thyroid hormones are normal and thyroid stimulating hormone (TSH) is above the reference values, subclinical hypothyroidism (sHT) is characterized (2). It is estimated that the prevalence of sHT in the general population is around 4% to 10%, which is higher among women (3).

The sHT patients may present signs and characteristic symptoms of hypothyroidism such as: fatigue, weight gain, dyslipidemia, psychological disorders, cardiovascular disorders, and increased risk of coronary artery disease and mortality (3,4). The effects of hypothyroidism on health related quality of life (HRQoL) are well established (5–7), but the results are controversial in sHT (3,4).

There is evidence that sHT is associated to worse scores in HRQoL domains such as: functional capacity, health perception, vitality and emotional aspects (6–8). In contrast, some studies do not support these findings (9–11). In part, the divergence of results can be explained by the degree of change in TSH levels. It seems that worse HRQoL are observed in patients with TSH higher than 10 mIU/L (2,6), although recent cross-sectional study has not confirmed this hypothesis (11).

Conventional treatment of sHT is done through hormone replacement with levothyroxine **(L-**T_4_) that is associated with decreased clinical manifestations in various organs and systems (4,12). It is emphasized, however, that it has produced conflicting results in the improvement of HRQoL and at the symptoms of patients in different studies (8,13).

Exercise is associated to a better HRQoL in different populations (14,15). However, few studies have investigated the effects of exercise on HRQoL of patients with sHT (16). Thus, this study aimed at evaluating if exercise improves HRQoL in women with sHT. According to previous studies with subclinical disorders (16,17), exercise is taken as hypothesis that also improve HRQoL in sHT.

## SUBJECTS AND METHODS

### Subjects

A total of 55 female participants were included, aged 20-60 years old, which composed two study groups: sHT group is consisted of 22 women recruited in the Endocrinology Service of Hospital and Maternity Terezinha de Jesus of *Faculdade de Ciências Médicas e da Saúde de Juiz de Fora,* Brazil. The inclusion criteria for the sHT group were: show two doses, with a minimum interval of four weeks of serum TSH above the adopted reference upper limit (4.94 mIU/L) and level of T_4L_ within the reference range (0.70 to 1.48 ng/dL). The control group consisted of 33 euthyroid women with normal values of serum TSH, anti-TPO (antithyroid peroxidase antibody) and free T4 within the reference ranges for the used kits and absence of thyroid disease history. Others criteria adopted for both groups were: absence of comorbidities and no physical exercise program for at least three months. Patients with sHT or euthyroid participants with any chronic and/or cardiovascular disease, smokers, those who were using drugs or substances that could interfere in thyroid function, in heart rate or blood pressure, as well as those with musculoskeletal inability toperform physical exercises were excluded from the study. The study was approved by the local ethics committee (no. 0164/10), and all participating groups signed free informed term of consent prior to participation in the study.

### Study design

Initially a cross sectional study comparing patients with sHT and euthyroid women concerned to signs and symptoms of hypothyroidism and HRQoL was performed. Subsequently, only sHT patients were randomized to participate or not in the exercise program. The patients were followed in both prospective study groups without masking. A resting echocardiography and exercise cardiopulmonary test were performed for clinical assessment of cardiac structure and cardiorespiratory function before the prospective phase. Two patients with severe changes in blood pressure and heart rate during the exercise test were excluded from randomization. Patients who participated in exercise training (n = 10) were denominated sHT-Tr, while those who did not participate in exercise training (n = 10) were denominated sHT-Sed. Exercise training consisted of aerobic activities, three times a week, for 16 weeks, supervised by the authors. The sHT-Sed patients were instructed for maintaining their usual daily life activities. After four months of intervention or observation, the tests conducted at the beginning of the study were repeated.

### Hormonal measures, level of physical activity and anthropometric measures

The TSH, T_4_L and anti-TPO levels were measured by third generation chemiluminescenceimmunometric assay (BeckmanCoulter^®^, Access2^®^). The reference values for TSH and free T_4_ were 0.35 to 4.94 mUI/mL and 0.70 to 1.48 ng/dL, respectively. The anti- TPO levels > 35 UI/mL were considered positive. The level of physical activity was assessed using the Baecke's Habitual Physical Activity Questionnaire, in its translated version and validated for Portuguese (18). In anthropometric assessment, body mass and height (Filizola scale with 0.1 kg and 10 mm precision, respectively), in order to calculate the Body Mass Index (BMI; kg/m^2^) were measured.

### Signs and symptoms of hypothyroidism - Clinical score

The specific signals and symptoms of hypothyroidism were evaluated by Billewicz scale modified (19). The scale consists of 12 clinical signals and symptoms of hypothyroidism: dry skin, rough skin, decreased sweating, weight gain, paresthesia, hoarseness, decreased hearing, constipation, periorbital edema, slow movements, cold skin, and slow Achilles reflex. A point (1) was assigned when the presence of signal or symptoms or zero (0) in his absence was found.

The maximum scale score is 12 points; scores lower than 3 are expected in euthyroid women; among 3 and 5, in sHT; and higher than 5 in hypothyroidism.

### Measure of health related quality of life

The SF-36 (Medical Outcomes Study 36 - Item Short-Form Health Survey) was used to assess the HRQoL of participants in their translated version and validated for Portuguese (20). The SF-36 is consisted of 36 items distributed in eight dimensions: functional capacity, physical aspects, pain, general health, vitality, social aspects, emotional aspects and mental health. The answers are presented in likert scale. The score of each domain ranges from 0 to 100 points and higher the score, higher HRQoL. The physical component was calculated by the average of the following scales: functional capacity, physical aspects, pain and general health; and the mental component: vitality, social aspects, emotional aspects and mental health.

### Exercise program

The exercise program consisted of aerobic activities with supervision of a physical education professional, involved in the study. The frequency of training was three times a week for 16 weeks. Each aerobic exercise session consisted of 60 minutes, divided into four phases: heating (5 minutes), ergometric bicycle (25 minutes), treadmill (25 minutes) and resting (5 minutes). The training was individually prescribed based on exercise cardiopulmonary test and maximum heart rate estimated by age (HR_max_ = 220 - age). The training intensity was controlled by HR between 65 and 75% of HR. The training was continuous and participants could walk, walk with inclination or run in treadmill. The HR was monitored during training sessions by HR monitor (Polar^®^). Blood pressure and rate of perceived exertion were measured every 10 minutes. After exercise session, stretching exercises were performed by participants and also stimulated to drink fluids.

### Statistical analysis

Descriptive analysis was presented as mean ± standard deviation or median (1^st^ quartile, 3^rd^ quartile). In the cross sectional study, comparisons between patients and euthyroid women were measured using Student's t tests or Mann-Whitney test. A two way repeated measures analysis of variance (ANOVA) (2X2) was performed to determine if significance differences between groups and time. If significant main effects or interactions were present a Bonferroni post hoc analysis was conducted. For the analysis of qualitative variables, Fisher's Exact Test was used. Relationship among quantitative variables was performed using Pearson's correlation test. As clinically relevant differences was considered the differences of at least 10 points on the scale of 0 to 100 (21). To assess the internal consistency of SF-36, the alpha Cronbach was used. All analyzes were carried out by using the statistical package SPSS 24.0 (IBM Corp., Armonk, NY). The significance level was set at P < 0.05.

## RESULTS

### Cross sectional study

The general characteristics and LQRH scores of women with sHT and euthyroid are shown in Table 1. In the sHT group, a total of 41% showed positive anti-TPO, while all euthyroid presented anti-TPO negative. There were no significant differences among groups regarding the variable potentials of confounding age (p = 0.85), body mass (p = 0.91), BMI (p = 0.94) and menopause status (p = 0.28). The sHT patients showed lower levels of physical activity (p < 0.001) and higher number of signals and symptoms (p = 0.02). Regarding LQRH, sHT patients showed lower scores on “functional capacity” domain (p = 0.02) compared to euthyroid. In other domains there were no significant differences observed between groups, however women with sHT showed consistently lower scores, except in “social aspects”. No relationship was found between TSH, signals and symptoms and life quality (p > 0.05). The internal consistency of 36 questions of SF-36 was satisfactory (Cronbach's alpha = 0.92). All domains also showed satisfactory coefficients (0.70 to 0.90). This means that the investigated sample reported high level of consistency in the answers to the survey questions.

**Table 1:** General characteristics and quality life scores of women with subclinical hypothyroidism (sHT) and euthyroid

	sHT (n = 22)	Euthyroid (n = 33)
Age (years)	39.4 ± 10.6	38.8 ± 8.7
TSH (mU/L)	5.58 (5.16-7.53)	2.10 (1.94-2.53)*
T_4_ (ng/dL)	0.95 (0.85-1.03)	0.98 (0.94-1.00)
Body mass (kg)	69.3 ± 17.0	69.8 ± 11.8
BMI (kg/m^2^)	26.4 ± 5.5	26.3 ± 5.0
Level of physical activity	7.1 ± 0.9	8.3 ± 1.3*
Signals and symptoms	3.5 ± 1.6	2.5 ± 1.0*
Domains and scores of SF-36		
Functional capacity	77.0 ± 23.0	88.8 ± 14.6*
Limitation by physical aspects	67.0 ± 40.4	81.1 ± 33.1
Pain	68.0 ± 26.6	68.4 ± 23.7
General health	71.1 ± 16.3	72.5 ± 19.2
Vitality	53.6 ± 23.0	58.0 ± 24.5
Social aspects	73.3 ± 29.2	70.1 ± 27.0
Limitation by emotional aspects	56.1 ± 45.3	57.6 ± 44.3
Mental health	66.4 ± 20.9	65.3 ± 24.5
Physical component	70.8 ± 20.3	77.7 ± 18.0
Mental component	62.3 ± 26.3	62.8 ± 25.4

Values: Mean ± SD and Median (Interquartile Range).

TSH: thyrotropin; T_4_: free thyroxine; BMI: body mass index; Level of physical activity measured by Baecke test; Signals and Symptoms of hypothyroidism measured by Zulewski test.

* Patients with sHT versus controls, significant difference p < 0.05.

### Randomized clinical trial

After randomization, there were no significant differences between the sHT-Tr and sHT-Sed groups related to age (p = 0.25), TSH (p = 0.85), T4 (p = 0.74), body mass (p = 0.35), BMI (p = 0.35), level of physical activity (p = 0.11), signals and symptoms (p = 0.53), menopause and all domains of life quality life (p > 0.05).

Analyses revealed a significant (group x time) interaction effect concerned functional capacity, general health, emotional aspects, psychological component, and physical component (Table 2). The sHT-Tr group showed improvement in these domains. On the other hand, the sHT-Sed group after four months of observation, showed no significant changes in all domains assessed by SF-36. Both groups showed no significant changes in signals and symptoms number (p > 0.05).

**Table 2:** General characteristics and quality life scores of patients with subclinical hypothyroidism before and after 4 months of exercise training (sHT-Tr) or observation (sHT-Sed)

	sHT-Tr(n=10)		sHT-Tr(n=10)		Group effect	Time effect	Interaction
	Baseline	4 months	Baseline	4 months			
Signals and symptoms	3.8 ± 2.0	3.3 ± 1.6	3.1 ± 1.4	3.1 ± 2.0	0.53	0.50	0.50
Domains and components of SF-36								
Functional capacity	73.0 ± 26.4	86.5 ± 9.4	85.0 ± 11.5	82.0 ± 17.7	0.58	0.21	0.049*
Limitation by physical aspects	65.0 ± 42.8	92.5 ± 12.1	75.0 ± 37.3	67.5 ± 44.2	0.55	0.37	0.12
Pain	67.7 ± 29.3	71.7 ± 17.6	72.7 ± 24.7	68.8 ± 24.3	0.92	0.99	0.36
General health	68.1 ± 17.4	83.0 ± 13.9	75.0 ± 16.2	69.5 ± 18.0	0.61	0.22	0.01*
Vitality	53.0 ± 30.7	70.0 ± 19.4	56.0 ± 16.3	56.5 ± 22.0	0.57	0.07	0.08
Social aspects	75.0 ± 27.0	87.5 ± 14.4	80.0 ± 28.4	77.5 ± 25.5	0.80	0.30	0.13
Limitation by emotional aspects	43.3 ± 49.8	90.0 ± 22.5	76.7 ± 35.3	63.3 ± 39.9	0.81	0.12	0.01*
Mental health	64.4 ± 26.5	76.4 ± 17.4	68.8 ± 17.1	68.0 ± 22.4	0.82	0.13	0.08
Physical component	68.5 ± 25.7	83.4 ± 6.6	76.9 ± 12.9	72.0 ± 20.2	0.82	0.29	0.04*
Mental component	59.0 ± 30.6	81.0 ± 14.4	70.4 ± 20.9	66.4 ± 24.3	0.86	0.09	0.02*

Values: Mean ± SD.

TSH: thyrotropin; T_4_: free thyroxine; BMI: body mass index; Level of physical activity measured by Baecke test; Signals and symptoms of hypothyroidism measured by Zulewski test.

* Significant difference p < 0.05, ANOVA 2x2.

## DISCUSSION

This study compared the HRQoL of women with sHT and the euthyroid and assessed the impact of physical exercise on this outcome. The main findings were: 1) sHT is associated to a worse perception of HRQoL; 2) Women with sHT showed improvements in multiple assessed domains of HRQoL after aerobic exercise training during four months.

In the cross sectional study, it was found that patients with sHT showed more signals and symptoms and lower functional capacity compared to control euthyroid group, what has been mentioned previously in other studies (6–8). Besides that, in our study, there was a greater presence of signs and symptoms in sHT patients compared to the control group. Similar results were beforehand observed (13). Previously, a study reported that the presence of signals and symptoms of thyroid dysfunction may be related to decreased quality of life (11). But it is difficult to distinguish sHT from the euthyroid only by signals and symptoms (22). This could explain the lack of difference in quality of life comparing these two groups in some studies (11).

Regarding the quality of life, there is controversy concerned to the results of studies involving patients with sHT (3). In our study, a worse quality of life in women with sHT was observed, mainly through the reduction of functional capacity compared to the euthyroid ones. Corroborating our results, there are studies that observed lower scores of functional capacity in women with sHT, associated with the worst perceptions of health, vitality and emotional aspects (6–8).

An earlier research found that patients with sHT showed lower scores in physical and psychological aspects and greater complaints related to fatigue, which cause damage to their daily activities (5). According to these authors, the decreased quality of life negatively influences mood and enhance anxiety and depression rates in sHT. In addition, studies showed lower functional capacity, general health and physical aspect (6) and worst health perception by patients with sHT, especially in the vitality factor, related to the psychological aspect (8). However, studies on Italy (9), Australia (10) and Netherlands (11) populations prove no worsening in quality of life in patients with sHT.

Studies have earlier identified worst quality of life in sHT (9,11). The results could be related not for disease diagnosis itself, but by the fact that patients are labeled as “sick” or their conscience are sick. Quality of life is a multidimensional and subjective construct, difficult for defining and systematization, which makes complex its operationalization (23). This, in part, could explain the divergent results reported in previous studies. Furthermore, another explanation would be no standardization of the criteria for assessing quality of life (24).

In the follow-up study, exercise training improves HRQoL after four months of intervention. There were increased domain scores for functional capacity, general aspects of health, emotional aspects and psychological and physical component. After 4 months of training, the patients had functional capacity values, general health, emotional aspects and higher mental and physical component of the normative values of Brazilian healthy women of the same age (25). Similar benefits were observed by other researchers (16), who conducted a study which evaluates the influence of a medium impact exercise program in relation to quality of life and cardiorespiratory fitness of women with sHT. Participants were subjected to a program that consisted of activity for 3 weekly sessions of 60 minutes during 12 weeks. After 12 weeks of intervention, women showed improvement in quality of life, through a higher score in most domains of SF-36, especially in relation to vitality, general health, social aspects of health and mental health. Furthermore, participants who were subjected to exercise program increased their cardiovascular fitness. In patients with subclinical hyperthyroidism it was found improvement in relation to the disease symptoms, especially in the perception of fatigue after 12 weeks of aerobic training (17).

It is unknown the actual mechanism responsible for the psychological effects of physical exercise, although it is recognized that it is an interaction of psychophysiological factors. In patients with coronary artery disease, for example, poor quality of life is associated to reduced exercise ability (14,15) and more tendency to fatigue (15). It is known that physical exercise is the main intervention used for physical fitness improvement and that it is positively related to quality of life (14,15,23,26). A systematic review to assess the association between physical activity and quality of life found that, in cross-sectional and longitudinal studies, the highest level of physical activity was related to a better perception of quality of life in apparently healthy adults or in different conditions of disease, regardless of sex (23). Moreover, a meta-analysis concluded that individuals with chronic diseases who received intervention to increase the level of physical activity improved their quality of life and that those who received supervised interventions showed the best result (24).

In clinical practice, the decision for whether or not treating the patient with sHT is connected to observed signals and symptoms, including patient complaints (3). Treatment with levothyroxine is generally associated with reduced pain, improvements in overall health and physical aspects (13), but not necessarily with better quality of life (27,28). In the present study, from the clinical point of view, the improvement observed in the quality of life of patients after physical training was moderate to high magnitude (> 10 points in SF-36 scales) (21). Thus, it is plausible to speculate that the improvement in the quality life of patients with sHT may be due to increased level of physical activity, physical fitness improvement and reduced signals and symptoms. Therefore, physical activity can be used as a strategy to improve health perception of these patients and should be encouraged by doctors and stimulate as a key element for the adoption of a healthy lifestyle for patients with sHT. Furthermore, the assessment of quality of life should be used in the diagnostic of these patients.

It is known that most patients with sHT tend to normalize spontaneously the TSH, especially those with TSH < 10 mIU/l. This normalization has no a well - defined pattern, although most patients may normalize their TSH levels between 6 and 18 months (29). In this same study, more than half of the patients had negative anti-TPO, and the changes in TSH were not correlated to the presence of anti-TPO (27). In this study, all patients practically showed TSH < 10 mIU/l. The effects of physical training on thyroid hormones are still a matter of debate in the literature and should be investigated in further studies. As limitations of the study, include the small sample size and the performed research only with women, not allowing, thus, expanding the results for male individuals.

In conclusion, the results suggest that women with subclinical hypothyroidism tend to have consistently lower scores on domains of quality of life compared to euthyroid women. However, physical exercise has been able to adjust these losses and therefore, should be encouraged in this group of patients. Further studies are necessary to better understand the optimal dose and the type of exercise would be more efficient to improve quality of life in women with sHT.
